# TGF*β* Signaling in Tumor Initiation, Epithelial-to-Mesenchymal Transition, and Metastasis

**DOI:** 10.1155/2015/587193

**Published:** 2015-03-25

**Authors:** Panagiotis Papageorgis

**Affiliations:** Department of Health Sciences, Program in Biological Sciences, European University Cyprus, 6 Diogenes Street, Engomi, 1516 Nicosia, Cyprus

## Abstract

Retaining the delicate balance in cell signaling activity is a prerequisite for the maintenance of physiological tissue homeostasis. Transforming growth factor-beta (TGF*β*) signaling is an essential pathway that plays crucial roles during embryonic development as well as in adult tissues. Aberrant TGF*β* signaling activity regulates tumor progression in a cancer cell-autonomous or non-cell-autonomous fashion and these effects may be tumor suppressing or tumor promoting depending on the cellular context. The fundamental role of this pathway in promoting cancer progression in multiple stages of the metastatic process, including epithelial-to-mesenchymal transition (EMT), is also becoming increasingly clear. In this review, we discuss the latest advances in the effort to unravel the inherent complexity of TGF*β* signaling and its role in cancer progression and metastasis. These findings provide important insights into designing personalized therapeutic strategies against advanced cancers.

## 1. Synthesis and Activation of TGF***β*** Family Members

The transforming growth factor-beta (TGF*β*) and TGF*β*-like molecules are members of a large superfamily of more than 40 secreted cytokines, including TGF*β*, bone morphogenetic proteins (BMPs), activins, nodal, lefty, myostatin, anti-müllerian hormone (AMH), and growth differentiation factors (GDFs). These pleotropic cytokines control numerous biological functions such as proliferation, apoptosis, embryonic patterning, stem cell maintenance, cell differentiation, migration, and regulation of the immune system. Unraveling the complexity that underlies their mode of action has remained challenging because these effects are known to be highly cell type-specific and context-dependent [1–3]. The three TGF*β* isoforms, TGF*β*1, TGF*β*2, and TGF*β*3, are the most widely studied members of the family, mostly because they are ubiquitously expressed and can influence the majority of tissue types. On the other hand, the expression of other cytokines is limited to only a few tissues, such as myostatin, or particular developmental stages, such as the AMH [3, 4]. The TGF*β* molecules are initially synthesized in an inactive pro-TGF*β* form, which consists of TGF*β* associated with latency associated proteins (LAPs). The TGF*β* large latent complex (LLC) consists of the LAPs and the latency-TGF*β*-binding proteins (LTBPs) assembled together with disulfide bridges between specific cysteine residues [5–8]. In turn, the LLC is covalently associated to the extracellular matrix (ECM) via the N-terminal region of LTBPs [9, 10]. The presence of the TGF*β* ligand within the LLC complex maintains the cytokine in an inactive form by preventing the interaction with its receptors [11]. TGF*β* can be activated in different ways. First, LAPs may undergo conformational changes induced by thrombospondin-1 (TSP-1) [12, 13] followed by cleavage mediated by proteases, including convertases, plasmins, or matrix metalloproteases (MMPs) [14–16]. Secondly, alpha v beta 6 (*α*_v_ *β*_6_) integrin, which becomes upregulated in response to wounding or inflammation, binds and activates latent TGF*β* [17]. Furthermore, TGF*β* can be activated by low pH levels in the local environment [18] or upon irradiation-induced reactive oxygen species (ROS) production [19]. Finally, mechanical contraction of myofibroblasts in the stroma can further activate latent TGF*β* [20]. All these mechanisms result in the release of active TGF*β* which can bind TGF*β* receptors and propagate downstream signaling events. Overall, the bioavailability of active TGF*β* ligand is greatly dependent on the maturation processes described above.

## 2. Sensing and Propagating TGF***β*** Signals

The SMAD family of proteins, comprised of eight structurally related human proteins, are the major effector molecules responsible for transducing intracellular signaling initiated by the TGF*β* superfamily of cytokines [21–25]. Smads can be categorized in three functionally distinct groups: the receptor activated Smads (R-Smads), which include Smad1, Smad2, Smad3, Smad5, and Smad8; the common mediator Smad (co-Smad), Smad4; and the inhibitory Smads (I-Smads), Smad6 and Smad7 [25, 26]. They all have similar sizes and range in molecular weights from 42 to 60 kDa. Structurally, the R-Smads and the co-Smad consist of two MAD homology (MH) domains, MH1 and MH2, located at the amino- and carboxy-terminals ends of the protein, respectively, and are separated by a proline-rich acidic linker region. On the other hand, the I-Smads lack the MH1 domain [27–29]. While the MH2 domain is involved in protein complex formation, as well as transcriptional activation and repression, the MH1 domain exhibits DNA binding activity [27, 30]. In the absence of TGF*β* ligand, the Smad proteins remain inactive because the MH1 and MH2 domains interact with each other resulting in their functional autoinhibition. TGF*β* stimulation induces conformational changes to relieve this inhibition allowing the MH2 domain of R-Smads to interact with the TGF*β* receptors [28, 31].

The TGF*β* isoforms transduce signaling via three types of TGF*β* receptors: TGF*β*RI, TGF*β*RII, and TGF*β*RIII. To date, seven TGF*β*RIs, five TGF*β*RIIs, and two TGF*β*RIIIs have been characterized: TGF*β*RIs include activin-like receptors 1–7 (ALK1–7); TGF*β*RIIs include the TGF*β*RII, BMPRII, ACTRII, ACTRIIB, and AMHRII; and betaglycan and endoglin belong to the TGF*β*RIIIs [32]. While TGF*β*RI and TGF*β*RII have been extensively studied during the last decade, the roles of TGF*β*RIII in physiology and disease have recently started to emerge. Betaglycan is considered to function as a coreceptor for TGF*β* superfamily, primarily to enhance activin/inhibin signaling [33]. On the other hand, endoglin appears to be predominantly expressed in endothelial cells and acts to control angiogenesis [34–36]. However, in most tissues, TGF*β* ligands function through heterotetrameric complex formation between two TGF*β*RIs and two TGF*β*RIIs. Both of these receptors exhibit Ser/Thr kinase activity but appear to have distinct roles; TGF*β*RIIs are able to activate the receptor complex while TGF*β*RIs propagate signaling to the R-Smads [37, 38]. TGF*β*RII-ALK5 complex formation can transduce signals from all three TGF*β* isoforms in multiple tissues, whereas TGF*β*RII can specifically associate with ALK1 in endothelial cells and with ALK2 in cardiovascular tissues [39]. While ALK5 is the predominant type of TGF*β*RI which functionally transduces TGF*β* signals, ALK2 and ALK4 are the ones which can bind activin with high affinity [38]. Also, alternative heteromeric receptor-ligand complexes can regulate distinct R-Smad family members. For example, ALK5 activates Smad2 and Smad3 (canonical TGF*β*/Smad-dependent signaling pathway) whereas ALK2, ALK3, and ALK6 activate Smad1, Smad5, and Smad8 (BMP signaling pathway) [40–46].

### 2.1. Smad-Dependent Signaling

**Figure 1:**
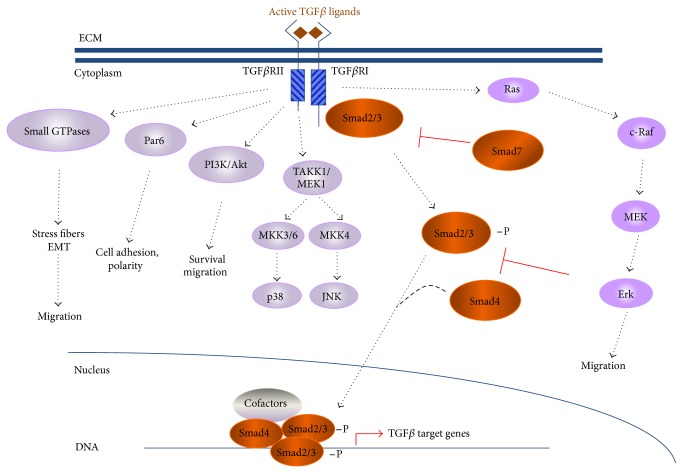
Smad-dependent and independent TGF*β* pathways. Active TGF*β* ligands initiate signaling by binding to TGF*β*RIs and TGF*β*RIIs. TGF*β* receptors exhibit kinase activities that are necessary for transducing canonical TGF*β* signaling by phosphorylating Smads2/3. Activated R-Smads can form a heterotrimeric complex with Smad4 which associates with other cofactors in the nucleus to regulate the expression of TGF*β* target genes. Furthermore, downstream signaling can also be transduced via auxiliary pathways such as various brunches of the Mek/Erk, the Rho-like GTPases, and the PI3K/Akt and the p38/MAPK pathways to modulate biological responses including epithelial-to-mesenchymal transition, cell adhesion, migration, and survival.

Mechanistically, all TGF*β* isoforms initiate signaling in a similar manner. The active TGF*β*1 ligand initially binds TGF*β*RII followed by recruitment of the ALK5 (TGF*β*RI) at the plasma membrane. With the heteromeric receptor-ligand complex formed, TGF*β*RII phosphorylates TGF*β*RI in a conserved Glycine-Serine (GS)-rich domain [47] leading to the dissociation of the inhibitory FKBP12 protein from TGF*β*RI [48]. This conformational switch allows activated TGF*β*RI to interact with R-Smads (Smad2/3) through their MH2 domain [49] resulting in their phosphorylation at the conserved SSXS C-terminal motif [31, 50]. SARA (Smad anchor for receptor activation) is a FYVE domain-containing protein which plays a central role in recruiting R-Smads to the activated TGF*β*RI to facilitate receptor-mediated phosphorylation. It preferentially associates with unphosphorylated Smad2 and is released upon Smad2 phosphorylation by TGF*β*RI [51]. This phosphorylation event triggers the formation of a heterotrimeric complex between phosphorylated R-Smads (Smad2/3) and co-Smad (Smad4), which can translocate into the nucleus to modulate gene expression (Figure 1) [3]. Smads act as transcription factors in cooperation with other coactivators, such as CBP/p300, P/CAF, SMIF, FoxO, Sp1, c-Jun/c-Fos, Sertad1, or corepressors, such as E2F4/5-p107, ATF3, TGIF, Ski, SnoN, FoxG1, EVI1, and CTBP [50, 52–68]. Furthermore, Smads can also indirectly regulate gene expression by controlling epigenetic processes, such as chromatin remodeling [69, 70] or by maintaining promoter DNA methylation, which is critical in silencing epithelial gene expression in cells that have undergone epithelial-to-mesenchymal transition (EMT) [71].

Importantly, the activity of TGF*β* signaling is balanced by a negative feedback loop mediated by the inhibitory Smad7, a major target gene of the pathway [72]. Under basal conditions, Smad7 resides in the cell nucleus but it translocates to the cell membrane upon TGF*β*-induced receptor complex formation [73]. Binding of Smad7 to the activated TGF*β* receptor complex inhibits propagation of downstream signaling by blocking interactions between the R-Smads and the activated receptors [74]. Furthermore, Smad7 can interact with the E3-ubiquitin ligases Smurf1 or Smurf2 in the nucleus. Upon TGF*β* activation, the Smad7-Smurf complex translocates to the plasma membrane where Smurf induces ubiquitination and proteasomal degradation of the TGF*β* receptors [75, 76]. Furthermore, in some cases, Smad7 can inhibit TGF*β*-mediated transcriptional events by directly binding to DNA, thus antagonizing the formation of a functional Smad-DNA complex [77].

It has also been shown that the R-Smads can regulate some cellular functions by partnering with factors other than Smad4. For example, TIF1*γ* (transcription intermediate factor 1*γ*) is able to compete against Smad4 for binding to Smad2/3 and plays a critical role in controlling erythroid differentiation [78]. Moreover, Smads2/3 can interact with I*κ*B kinase *α* (IKK*α*), in a Smad4-independent manner, to regulate the expression of Mad1, an antagonist of the Myc oncogene, and control keratinocyte differentiation [79]. These findings are consistent with the notion that Smad4 is essential for many, but not all, TGF*β*-regulated Smad-dependent cellular responses.

### 2.2. Smad-Independent Signaling

Several studies have clearly demonstrated that TGF*β* can also employ Smad-independent pathways as downstream effectors [80]. TGF*β* induces activation of Erk signaling in multiple tissues including epithelial and endothelial cells, as well as in breast and colorectal cancer cells to promote the dissolution of adherens junctions and cell migration [81–87]. This induction is either indirect via activation of TGF*α* and FGF autocrine loops [84, 85] or can be direct by Src-mediated or autophosphorylation of TGF*β*RII at Tyr residues [88, 89]. Moreover, phosphorylation of TGF*β*RI at Tyr residues activates ShcA to promote the formation of a ShcA/Grb2/Sos complex. Subsequently, this complex can activate Ras on the plasma membrane, which in turn transduces downstream signaling via c-Raf, MEK, and Erk [90]. TGF*β* can also promote activation of the JNK and p38-MAPK pathways to regulate apoptosis or cell migration, depending on cellular context [91–93], via MKK4 and MKK3/6, respectively [94, 95]. Further upstream, these MKKs can be phosphorylated by either TRAF6-mediated recruitment of TAK1 [96–99], or by two other MAPKKKs, MEKK1, and MLK3 [100, 101]. Furthermore, the PI3K/Akt pathway has been implicated in mediating some of cellular functions of TGF*β*. Studies have shown that TGF*β* can rapidly induce PI3K activation followed by phosphorylation of its effector Akt to promote EMT, cell migration, and survival [102, 103]. Mechanistically, the p85 regulatory subunit of PI3K appears to be constitutively bound to the TGF*β*RII and, upon TGF*β* stimulation, TGF*β*RI is recruited to the complex to activate PI3K and initiate downstream signaling [104]. The mammalian target of rapamycin (mTOR) acts as a major effector molecule of this pathway by controlling the phosphorylation of S6 kinase (S6K) and eukaryotic initiation factor 4E binding protein [105]. Activation of the mTOR pathway by TGF*β* has been shown to be critically important for regulating protein synthesis, cell size, and EMT [106]. Finally, certain TGF*β* functions, such as rearrangement of cytoskeletal organization, cell polarity, and cell migration, are mediated by the Rho-like family of small GTPases [107]. TGF*β* can rapidly activate the RhoA and Cdc42/Rac1 pathways, in a Smad2/3-independent manner, to promote EMT, actin polymerization, and formation of stress fibers (Figure 1) [108, 109]. Conclusively, this evidence strongly supports that Smad-independent pathways play critical roles in regulating TGF*β*-mediated cellular functions.

## 3. TGF***β*** Signaling in Cancer Initiation

It is unambiguously accepted that TGF*β* plays fundamental roles in carcinogenesis and tumor progression. However, numerous studies have clearly demonstrated that TGF*β* acts as a double-edge sword during this process. Initially, TGF*β* is able to suppress growth in normal and premalignant epithelial cells. However, upon accumulation of genetic and epigenetic alterations in tumor cells, it switches to promotion of a proinvasive and prometastatic phenotype, accompanied by a progressive increase in the locally secreted TGF*β* levels. The complexity of these functions is further increased due to the fact that these TGF*β* functions may vary depending on the type and genetic background of tissues [110–113].

### 3.1. Regulation of Cell Proliferation and Apoptosis

Numerous* in vitro* studies using human cells as well as data from animal models provided concrete evidence for the role of TGF*β* as a tumor suppressor in various normal tissues. It is well known that TGF*β* has a growth-inhibitory effect on normal epithelial [114], endothelial [115, 116], and neuronal cells [117] as well as on cells of the immune system, such as T-cells [118]. Under physiological conditions, the cytostatic role of TGF*β* is critical in order to prevent the generation of hyperproliferative disorders, such as fibrosis and cancer. Mechanistically, TGF*β* can induce the expression of genes involved in opposing proliferative cell responses during all phases of the cell cycle, but it is primarily implicated in G1/S phase transition events [119]. Genome-wide transcriptional profiling studies using normal human epithelial cell lines from mammary gland, skin, and lung have identified a common set of genes that are transcriptionally regulated by TGF*β* in order to mediate its cytostatic effects. This transcriptional program predominantly involves the activation of the G1/S phase checkpoint and cell cycle arrest by two main mechanisms. First, TGF*β* induces the expression of the cyclin-dependent kinase inhibitors* CDKN2B* (encoding p15/INK4B) [120],* CDKN1A* (encoding p21/Cip/Waf1) [121], and p27/Kip1 [122]. In mammary epithelial cells, the induction of expression and protein stability of p15 enhances the formation of p15-CDK4/6 complexes and therefore inhibits cyclin D1-CDK4/6 association [123]. During early G1 phase and in the absence of TGF*β*, cyclin D1-CDK4/6 complex formation is required for mitogen sensing and cell cycle progression through S phase. However, upon TGF*β*-mediated p15 upregulation, p15 binds CDK4 and/or CDK6, inhibiting their catalytic activity and preventing their association with cyclin D1, resulting in cell cycle arrest. TGF*β* can also inhibit G1/S phase progression by inhibiting the formation of cyclin E-CDK2 and cyclin A-CDK2 via induction of p21 and p27, which bind to these cyclin-CDK complexes and similarly cause their functional inactivation [124, 125]. The second mechanism by which TGF*β* inhibits cell cycle progression is by repressing the expression of the proliferation-inducing transcription factors c-Myc [126] and the family of inhibitor of DNA binding proteins ID1, ID2, and ID3 [57, 127]. In proliferating cells, c-MYC is recruited by the zinc-finger protein MIZ1 to the* CDKN2B* and* CDKN1A* gene promoters to suppress their transcription. Upon TGF*β* stimulation, c-MYC expression is downregulated and the suppression of CDKN2B/CDKN1A is relieved [128, 129]. Suppression of ID family members also contributes to the cytostatic effects by TGF*β*. The ID proteins are able to physically interact and inactivate the tumor suppressor retinoblastoma (Rb) protein to promote cell proliferation [130]. TGF*β* promotes the formation of an ATF3-Smad3/Smad4 complex to transcriptionally repress ID1 expression [57], while downregulation of ID2 is achieved indirectly via suppression of the ID2-inducer c-MYC [130].

The growth inhibitory effects of TGF*β* on epithelial tissues are also supported by gain or loss-of-function experiments using transgenic animal models. For examples, exogenous tissue-specific overexpression of TGF*β* in the epidermis decreased keratinocyte proliferation and protected mice from carcinogen-induced hyperplasia and skin tumorigenesis [131]. Similarly, transgenic expression of a dominant-negative form of the TGF*β*RII in the mouse epidermis blocked TGF*β*-induced growth inhibition [132]. In addition, Smad3-null mice exhibit increased keratinocyte proliferation and accelerated ability for wound healing [133]. Analogous findings were also reported for other tissues, such as the mammary gland and the colon. MMTV-driven overexpression of active TGF*β* in the mammary gland of transgenic mice resulted in the formation of a hypoplastic virgin mammary gland and impaired alveolar development during pregnancy [134, 135]. Conversely, overexpression of dominant-negative TGF*β*RII in the mouse mammary gland resulted in increased side-branching, hyperplasia, and sensitivity to carcinogens [136, 137], whereas overexpression of the same transgene in the colon reduced TGF*β*-mediated growth arrest [138].

In some cases, TGF*β* is also known to induce apoptosis in these tissues even though the molecular mechanisms of this process remain poorly understood. Despite the fact that the induction of TGF*β*-mediated apoptosis has yet to be established* in vivo*, studies using cell lines revealed a number of candidate proteins that may be implicated in this effect [139]. Initially, upregulation of the TGF*β*-inducible early response gene-1 (TIEG1) by TGF*β* was found to trigger apoptosis in pancreatic epithelial cells. Furthermore, TGF*β* was shown to promote apoptosis of hepatoma cells via a Smad-dependent upregulation of the death-associated protein kinase DAPK [140]. Moreover, the adaptor protein Daxx has been implicated in mediating TGF*β* apoptotic actions by enhancing JNK signaling [141]. Similarly, TGF*β* is able to induce GADD45b expression which in turn stimulates p38-MAPK signaling, followed by caspase-8 and Bad activation to promote apoptosis [142]. Furthermore, TGF*β* leads to ARTS translocation from the mitochondria to the nucleus where it physically interacts and suppresses the function of XIAP, a major inhibitor of apoptosis [143]. Finally, Bim was also shown to be another proapoptotic TGF*β* target, which activates Bax to promote caspase-dependent apoptosis [144].

It is important to highlight that the effects of TGF*β* in proliferation can be different, even opposing, depending on the tissue type. While TGF*β* inhibits proliferation of normal epithelial, endothelial, neuronal, and T cells, it can also enhance the proliferation of fibroblasts [114]. In fact, 30 years ago, the initial experiments that led to the discovery of TGF*β* and its naming as “transforming” growth factor were based on its ability to induce proliferation and transformation of fibroblasts [145]. This effect is mediated indirectly by TGF*β*-induced connective tissue growth factor (CTGF) secretion, which is responsible for stimulating fibroblast proliferation [146]. Nonetheless, in most normal tissues TGF*β* predominantly acts as an inhibitor of cell proliferation.

### 3.2. Smad Pathway Alterations in Human Cancers

One of the hallmarks of most cancer types is that the vast majority of cases exhibit insensitivity to TGF*β*-mediated growth inhibition. Studies in human tumors have shown that TGF*β* pathway components often become genetically inactivated in certain cancer types to explain, in part, the acquired insensitivity of TGF*β*-mediated growth control. Loss of function or truncating mutations in* TGF*β*RI*,* TGF*β*RII*,* SMAD2,* and* SMAD4* genes have been detected in colorectal, pancreatic, gastric, and prostate tumors [21, 147–151]. Furthermore, 18q21 chromosome loss, harboring the Smad4 gene, is observed in 60% of pancreatic and 30% of colorectal cancers [152–155]. Subsequent functional studies further elucidated the role of Smad signaling inactivation in pancreatic and colorectal cancer progression. Restoration of Smad4 expression in pancreatic cancer cell lines suppresses tumor growth and angiogenesis by decreasing VEGF levels [156]. Similarly, homozygous Smad4 deletion accompanied by TGF*β* overexpression induces VEGF expression via the MEK-Erk and p38 pathways in order to facilitate colon cancer progression and drug resistance [83].

Notably, these genetic alterations are not detected in all tumor types. For example, in breast cancers Smad gene mutations are rare [21, 150, 151] suggesting that additional mechanisms for acquiring resistance to TGF*β*-mediated growth inhibition also exist. It has been shown that activation of the Ras oncogene and its downstream target Erk leads to the phosphorylation of Smad1, Smad2, and Smad3 in their linker region, thus inducing their retention in the cytoplasm and promoting their ubiquitin-dependent degradation [157–159]. In addition, metastatic breast cancer cells, isolated from pleural fluids of patients, exhibit intact Smad pathway components but were found to be unresponsive to TGF*β*-mediated growth inhibition. In this study, the cytostatic responses to TGF*β* appeared to be dependent on the transcription factor C/EBP*β* which is essential for the induction of the cell cycle inhibitor p15/INK4b and the repression of c-MYC oncogene. Interestingly, cells from half of these patients overexpressed the dominant-negative C/EBP*β* isoform LIP, which is able to bind and inhibit the transcriptionally active C/EBP*β* isoform LAP in order to suppress TGF*β*-mediated growth inhibition [160]. Another mechanism that TGF*β* may exploit in order to switch from a tumor suppressive to a metastasis-promoting factor is through differential regulation of* ID1* gene. While ID1 expression is suppressed by TGF*β* in normal tissues, it was found to be induced in patient-derived metastatic breast cancer cells [161]. Importantly, high ID1 expression levels are correlated with relapse in patients with estrogen receptor negative (ER) breast tumors [162]. Also, the Tax oncoprotein, encoded by the Human T-cell leukemia virus type I (HTLV-I), is able to inhibit Smad-dependent transcription in T cells, thus contributing to the acquisition of resistance to growth inhibition [163]. Finally, the SKI and SKIL oncoproteins can interact with Smad3 and Smad4 to displace p300 and CBP from the active transcriptional complex in order to repress TGF*β*-mediated growth inhibition [164].

## 4. TGF***β*** in Epithelial-to-Mesenchymal Transition (EMT)

**Figure 2:**
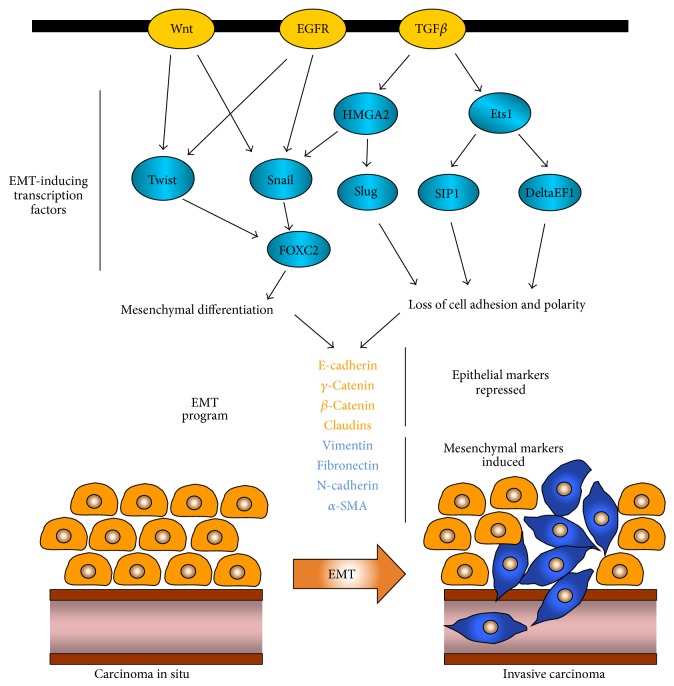
TGF*β* signaling in epithelial-to-mesenchymal transition. TGF*β* signaling mediated by Smad or non-Smad pathways can directly or indirectly induce the expression of different transcriptional “master regulators” of epithelial-to-mesenchymal transition. These factors, including Snail, Slug, ZEB1/delta EF1, and ZEB2/SIP1 are able to initiate a coordinated transcriptional network which results in suppression of epithelial and upregulation mesenchymal marker expression. As a result, epithelial cancer cells undergo dissolution of adherens and tight junctions along with dramatic remodeling of their cytoskeleton and acquire mesenchymal features. These fibroblast-like, spindle shaped tumor cells exhibit significantly enhanced migratory and invasive potential which allows them to enter the blood circulation through the basement membrane and initiate their metastatic dissemination to distal organs.

EMT is a vital process for morphogenesis during embryonic development and was initially appreciated primarily by developmental biologists. During the last decade, however, it has become apparent that EMT can be abnormally reactivated in adult tissues during pathological conditions such as cancer and fibrosis [165]. EMT involves the induction of an orchestrated, reversible transcriptional program in which well-organized, tightly connected epithelial cells transdifferentiate into disorganized and motile mesenchymal cells. This process is characterized by disruption of tight junctions between epithelial cells due to downregulation and delocalization of tight junction proteins zonula occludens 1 (ZO-1), occludin, and claudins. Similarly, adherens cell junction complexes containing E-cadherin, p120, *γ*-catenin, and *β*-catenin also undergo dissolution. This is followed by loss of apical-basal cell polarity, dramatic remodeling of the cytoskeleton, and the formation of actin stress fibers. Concomitantly, cells acquire mesenchymal features such as spindle-shaped, fibroblast-like morphology and express mesenchymal components including N-cadherin, vimentin, fibronectin, and alpha smooth-muscle actin [166, 167]. TGF*β* signaling plays an instrumental role in activating this transcriptional network by inducing the expression of several pleiotropically acting transcription factors, also known as “master regulators” of EMT. TGF*β*-induced factors include the Snail family of proteins Snail [168] and Slug [169] as well as the two-handed zinc finger factors ZEB1/deltaEF1 [170] and ZEB2/SIP1 [171] while the basic helix-loop-helix (bHLH) protein Twist [172] can be upregulated by Wnt, EGFR, or STAT3 signaling [173, 174]. Other EMT transcription factors, also induced by the TGF*β*-Smad pathway, such as HMGA2 [175] or Ets1 [176], act as upstream regulators in this network by upregulating the expression of Snail and ZEB family members, respectively. On the other hand, FOXC2 is a factor which functions downstream of Snail and Twist to promote EMT (Figure 2) [177]. In addition to these transcriptional mechanisms, recent studies indicate that overactive TGF*β*-Smad2 signaling further contributes to the establishment of an EMT phenotype bymaintaining the epigenetic silencing of key epithelial marker genes, such as E-cadherin, claudin-4, kallikrein-10, and cingulin. This appears to be mediated via Smad2-dependent regulation of DNA methyltransferase 1 (DNMT1) binding activity and DNA methylation of the corresponding gene promoter regions [71].

Therefore, one of the main mechanisms by which TGF*β* promotes cell migration, invasion, and metastasis is through induction of EMT. Studies have shown that TGF*β* stimulation of carcinoma-derived cell populations in culture can lead to the activation of this reversible process [45, 178, 179].* In vivo* studies have further shown that expression of TGF*β*1 in the skin of transgenic mice enhanced the conversion of benign skin tumors to carcinomas and highly invasive spindle-cell carcinomas [180]. Moreover, expression of a dominant-negative TGF*β*RII prevented squamous carcinoma cells from undergoing EMT in response to TGF*β in vivo* [181]. Acquisition of an EMT phenotype results in cells with diminished adhesive capacity that are highly migratory and invasive due to increased secretion of extracellular proteases. Therefore, EMT enhances intravasation of carcinoma* in situ* cells through the basement membrane in the circulation and facilitates extravasation at the distal tissues and formation of micrometastases in distal organs [165, 172, 182].

Besides Smads, other signaling molecules have also been implicated in TGF*β*-mediated EMT, including Erk, PI3K-Akt, RhoA, and cofilin [183, 184]. Induction of Erk and p38-MAPK phosphorylation by TGF*β* regulates the expression of genes involved in the remodeling of extracellular matrix and disruption of adherens and tight junctions to facilitate EMT [95, 185]. However, studies using Smad-binding defective TGF*β*RI constructs that can still mediate MAPK signal indicated that Smads are required for Erk-induced EMT process [95, 186]. Consistent with this evidence, other reports have demonstrated that cooperation between the TGF*β* and Ras-Raf-MAPK pathways is involved in promoting EMT [178, 179, 187, 188]. Additional molecular evidence to support this synergistic effect resulted from studies showing that, under the influence of oncogenic Ras, formation of a mutant p53/Smad complex empowers TGF*β*-induced metastasis by opposing p63 activity [189].

Finally, the microRNA 200 family members miR-200 and miR-205 have been shown to inhibit the E-cadherin repressors ZEB1 and ZEB2 to suppress EMT and promote an epithelial phenotype. Interestingly, loss of expression of these noncoding RNAs is observed in breast tumors and may facilitate EMT, invasion, and metastasis [190–193]. Furthermore, TGF*β* suppresses miR-203 expression leading to upregulation of its target SLUG in order to promote EMT and metastasis [194]. In contrast, upregulation of miR-21 in some tumor types facilitates TGF*β*-induced EMT and cancer cell migration [195]. In summary, it is becoming increasingly clear that TGF*β* signaling controls a complex network of interconnected pathways to regulate EMT and, therefore, the metastatic properties of cancer cells.

## 5. TGF***β*** and ‘‘Cancer Stem Cells” (CSCs)

Evidence that emerged more than a decade ago strongly suggested that a subset of undifferentiated breast cancer cells that exhibit a CD44^high^/CD24^low^ cell surface marker expression pattern possess stem cell-like properties and have a strong ability to initiate tumor formation, even at very low numbers [196]. According to the “cancer stem cell hypothesis,” stem-like cancer cells are thought to represent a subpopulation of tumor cells that also promote cancer metastasis and resistance to therapy [197, 198]. Interestingly, TGF*β*-induced EMT has been shown to generate cancer cells with stem-like properties through autocrine and paracrine loops [199, 200]. Therefore, aberrant activity of the TGF*β* signaling pathway, in the vast majority of solid tumors, could be functionally linked to the development and maintenance of cancer stem cells, further supporting the notion that this pathway may represent an attractive target for cancer therapy. However, despite the numerous reports using experimental approaches showing the significance of EMT in cancer progression, the detection of this phenomenon and its importance in clinical histopathological samples has remained challenging. Recent findings convincingly demonstrated that circulating tumor cells (CTCs) from breast cancer patients exhibit dynamic changes between epithelial and mesenchymal characteristics during the course of therapy. Interestingly, the mesenchymal phenotype in CTCs correlated with expression of TGF*β* and FOXC1 as well as with disease progression [201].

## 6. TGF***β*** in the Tumor Microenvironment

In many tumor types, excessive TGF*β* secretion is often detected locally, in the microenvironment surrounding the tumor and within the stroma to promote invasion of the leading tumor front and facilitate metastasis [202–205]. TGF*β* can be derived either from cancer cells [206] or from tumor infiltrating stromal cells, such as fibroblasts, macrophages, and leukocytes, as well as mesenchymal and myeloid precursor cells [207]. Also, TGF*β* can be stored in the extracellular matrix (ECM) of the bone, becoming biologically activated during development of osteolytic metastatic lesions [208].

Within the tumor microenvironment, TGF*β* exhibits a dynamic interaction with various stromal components. It plays a major role in the differentiation of mesenchymal progenitor cells into fibroblasts followed by conversion into myofibroblasts [209]. The latter, characterized by alpha-smooth muscle actin (*α*-SMA) expression, are highly contractile cells that further contribute to the secretion of TGF*β* in the microenvironment. When stimulated by TGF*β* in an autocrine or paracrine fashion, myofibroblasts produce extracellular matrix components, such as collagen, fibronectin, tenascin, osteopontin, osteonectin, and elastin, which create a desmoplastic ECM [210]. In this environment, myofibroblast contraction stimulates the release of active TGF*β* from its latent form that is stored in the ECM [20, 211].

Furthermore, TGF*β* elicits strong immunosuppressive effects by inhibiting the functions of different immune cell types. It has long been known that TGF*β* inhibits the proliferation of and suppresses the antitumor functions of CD4+ or CD8+ T cells, both* in vitro* and* in vivo* [212, 213]; it is also capable of inducing apoptosis in B-cells [214]. TGF*β* inhibits T-cell activation by suppressing antigen-presenting dendritic cells, which are responsible for the maturation and effective stimulation of T cells during immune responses [215]. In addition, TGF*β* blocks the production of IFN*γ* by natural killer (NK) cells to weaken their ability to recognize and eliminate cancer cells [212]. Finally, TGF*β* can promote tumor growth by inducing polarization of macrophages and neutrophils from the cancer cell-attacking type 1 to the type 2, which exhibits significantly reduced effector function and produces inflammatory cytokines, like IL-6, IL-11, and TGF*β* [216, 217]. These studies collectively establish a critical role for TGF*β* in suppressing host immune system to facilitate cancer progression.

## 7. Priming for Metastasis and Colonization

Dissemination of cancer cells is thought to represent a nonrandom, biologically active process which can be driven by specific genes, depending on the specific organs of metastasis [162, 218–220]. TGF*β* has been shown to play a critical role in these processes, such as promoting breast cancer metastasis to the bone via the Smad pathway [221]. Also, TGF*β* in the tumor microenvironment is able to prime breast cancer cells for pulmonary metastasis by inducing angiopoietin-like 4 (ANGPTL4) secretion which facilitates retention of cancer cells to the lungs [161].

Once cancer cells extravasate to a secondary tissue, they initially form micrometastatic lesions. However, since colonization of tumor cells in distal organs is a highly inefficient process, often described as the “rate-limiting step” of metastasis, cancer cells can remain in a dormant state which may last up to several years in cancer patients. Dormancy of cancer cells is a poorly understood condition which is largely responsible for local recurrence and metastatic growth even years or decades after therapy [222, 223]. While EMT is critical for the initiation of the metastatic cascade, colonization to distal tissues requires the reversal of this process which is described as mesenchymal-to-epithelial transition (MET) [224]. TGF*β* has been shown to play a role during metastatic colonization by inducing ID1 expression only in cells that have already undergone EMT. In turn, ID1 upregulation promotes MET by suppressing Twist1 expression [225]. Interestingly, a recent study has identified that TGF*β* may promote metastasis and organ colonization of hepatocellular carcinoma by upregulating the long noncoding RNA IncRNA-ATB [226]. Besides TGF*β*, signaling via the closely related member of the superfamily bone morphogenetic growth factor (BMP) has been linked with metastatic colonization. Inhibition of BMP signaling by the secreted antagonist Coco was found to reactivate breast cancer cells at lung metastatic sites and promote their colonization [227].

## 8. Conclusions and Future Perspectives

It is unambiguously accepted that TGF*β* signaling plays crucial roles during cancer progression and represents an attractive target for antimetastatic therapy. Several different promising therapeutic approaches are currently being tested in clinical trials or are still under preclinical investigation to evaluate their efficacy as antimetastatic molecules. These include blockers of TGF*β* activation, ligand traps, neutralizing antibodies against TGF*β*-receptor interaction, antisense oligonucleotides, or inhibitors of TGF*β* receptor kinase activity [228]. However, since TGF*β* exerts complex functions acting both as a tumor suppressor and a metastasis-promoting cytokine depending on cellular context, inhibition of TGF*β* signaling as a therapeutic strategy must be approached with caution. The future use of such TGF*β* signaling modulating drugs in the clinic must be carefully assessed, considering their effects on cancer cells and on cells of the tumor microenvironment in addition to potentially deleterious effects of these strategies on normal tissues.
